# Nanovesicular Mediation of the Gut–Brain Axis by Probiotics: Insights into Irritable Bowel Syndrome

**DOI:** 10.3390/biology13050296

**Published:** 2024-04-25

**Authors:** Radha Santonocito, Letizia Paladino, Alessandra Maria Vitale, Giuseppa D’Amico, Francesco Paolo Zummo, Paolo Pirrotta, Samuele Raccosta, Mauro Manno, Salvatore Accomando, Francesco D’Arpa, Francesco Carini, Rosario Barone, Francesca Rappa, Antonella Marino Gammazza, Fabio Bucchieri, Francesco Cappello, Celeste Caruso Bavisotto

**Affiliations:** 1 Section of Human Anatomy, Department of Biomedicine, Neuroscience and Advanced Diagnostics (BIND), University of Palermo, 90127 Palermo, Italy; radha.santonocito@unipa.it (R.S.); letizia.paladino@unipa.it (L.P.); alessandramaria.vitale@unipa.it (A.M.V.); giuseppa.damico01@unipa.it (G.D.); francescopaolo.zummo01@unipa.it (F.P.Z.); francesco.carini@unipa.it (F.C.); rosario.barone@unipa.it (R.B.); francesca.rappa@unipa.it (F.R.); antonella.marinogammazza@unipa.it (A.M.G.); fabio.bucchieri@unipa.it (F.B.); or francesco.cappello@iemest.eu (F.C.); 2 Euro-Mediterranean Institute of Science and Technology (IEMEST), 90139 Palermo, Italy; paolopirrotta@iemest.eu; 3 Cell-Tech Hub, Institute of Biophysics, National Research Council of Italy, 90146 Palermo, Italy; samuele.raccosta@ibf.cnr.it (S.R.); mauro.manno@cnr.it (M.M.); 4 Department of Health Promotion, Mother and Childcare, Internal Medicine and Medical Specialities “G D‘Alessandro”, PROMISE, University of Palermo, 90127 Palermo, Italy; salvatore.accomando@unipa.it; 5 Department of Surgical, Oncological and Stomatological Disciplines, DICHIRONS, University of Palermo, 90127 Palermo, Italy; francesco.darpa@unipa.it

**Keywords:** gut microbiota, probiotics, stress, heat shock proteins, tryptophan metabolism, extracellular vesicles, gut–brain axis

## Abstract

**Simple Summary:**

In this study, we studied the potential health benefits of probiotic supplements in individuals with chronic diarrhea. Dysbiosis, resulting from factors like poor diet or stress, can lead to various systemic diseases. Probiotics are known for stabilizing gut microbiota and alleviating gastrointestinal issues, such as irritable bowel syndrome (IBS). We focused on the tryptophan pathway’s role in regulating serotonin levels and its impact on host physiology and behavior. Nanovesicles isolated from subjects’ plasma before and after 60 days of probiotics consumption showed enhanced levels of Tryptophan 2,3-dioxygenase 2 (TDO 2), suggesting a potential role in the gut–brain axis. In vitro experiments demonstrated the probiotics’ cytoprotective effect against H_2_O_2_-induced stress, reducing heat shock protein 60 kDa levels and preserving intestinal integrity. Moreover, the probiotics increased TDO 2 and serotonin receptor expression. These results provide evidence for the role of nanovesicles in mediating the gut–brain axis and offer insights into potential therapeutic avenues for neurological disorders.

**Abstract:**

Background: Dysbiosis, influenced by poor diet or stress, is associated with various systemic diseases. Probiotic supplements are recognized for stabilizing gut microbiota and alleviating gastrointestinal issues, like irritable bowel syndrome (IBS). This study focused on the tryptophan pathways, which are important for the regulation of serotonin levels, and on host physiology and behavior regulation. Methods: Nanovesicles were isolated from the plasma of subjects with chronic diarrhea, both before and after 60 days of consuming a probiotic mix (Acronelle^®^, Bromatech S.r.l., Milan, Italy). These nanovesicles were assessed for the presence of Tryptophan 2,3-dioxygenase 2 (TDO 2). Furthermore, the probiotics mix, in combination with H_2_O_2_, was used to treat HT29 cells to explore its cytoprotective and anti-stress effect. Results: In vivo, levels of TDO 2 in nanovesicles were enhanced in the blood after probiotic treatment, suggesting a role in the gut–brain axis. In the in vitro model, a typical H_2_O_2_-induced stress effect occurred, which the probiotics mix was able to recover, showing a cytoprotective effect. The probiotics mix treatment significantly reduced the heat shock protein 60 kDa levels and was able to preserve intestinal integrity and barrier function by restoring the expression and redistribution of tight junction proteins. Moreover, the probiotics mix increased the expression of TDO 2 and serotonin receptors. Conclusions: This study provides evidence for the gut–brain axis mediation by nanovesicles, influencing central nervous system function.

## 1. Introduction

Probiotic use is gaining traction and constitutes a significant and expanding sector within the commercial market of dietary supplements and is heralded for its potential to confer various health benefits to humans [1,2,3,4]. Among the myriad of claimed advantages, probiotics are renowned for their role in stabilizing the gut microbiota, mitigating gastrointestinal disorders, such as irritable bowel syndrome (IBS), and modulating the host immune system [5,6,7,8]. The muco-microbiotic (MuMi) layer of the intestine houses the microbiota along with all the soluble factors that allow communication between the microbial elements and the epithelial cells of the intestinal mucosa [9,10]. These microorganisms have co-evolved with the host to perform numerous beneficial functions, ranging from simple food fermentation to extensive effects on immune system development, stress responses, and behavior [11,12,13]. As a result, the complex community of bacteria has a significant impact on host health [11]. A poor diet characterized by excessive consumption of food high in saturated fats, added sugars, processed foods, and low in essential nutrients such as fruits, vegetables, whole grains, and lean proteins as well as the inappropriate use of antibiotics or stress can lead to an imbalance of the gut microbiota. The disruption in the composition and function of the microbiota is a condition called dysbiosis, which can occur when there is a decrease in beneficial microorganisms and an increase in harmful microorganisms [3,4]. A state of protracted dysbiosis underlies the development of altered intestinal permeability syndrome, known as “leaky gut” [14,15], a condition characterized not only by the presence of bothersome symptoms, such as abdominal pain, constipation, and diarrhea, but also associated with a dangerous state of persistent inflammation and activation of the immune system that can lead to the onset of inflammatory and metabolic diseases involving both the proximal intestine (as in the case of chronic inflammatory diseases, IBD) and distant organs (as in the case of some neurodegenerative and neuropsychiatric disorders, including depression) [14,16,17]. Metabolite production by both the commensal community and the host actively contributes to the proper communication underlying homeostasis and includes metabolites produced by the intestinal flora, such as short-chain fatty acids (SCFA), indole, lactic acid, and host metabolites, such as hormones, vitamins, enzymes, lipids, and amino acids [18,19]. Among these, tryptophan is an essential amino acid found in many foods of both animal and plant origin that performs numerous physiological functions, including protein synthesis [20]. It is also a biosynthetic precursor of several neurologically active compounds, such as the hormone melatonin and the neurotransmitter serotonin, which is implicated in mood (which is why we often refer to the microbiota as psychobiota). One tryptophan pathway that has only recently interested the scientific community is the kynurenine pathway due to its influence on the immune system, inflammatory status, and neurological conditions. In fact, most of the degradation of tryptophan occurs through this pathway by the enzyme Tryptophan 2,3-dioxygenase 2 (TDO 2), which takes place in several tissues, including the intestinal mucosa [21]. In this study, we assessed the presence of the enzyme TDO 2 in nanovesicles isolated from the plasma of subjects with intestinal malabsorption disorders and chronic diarrhea who underwent a 60-day administration of a probiotic mix. This mix consisted of *Lactobacillus Salivarius* LS33 (*L. Salivarius*), *Lactobacillus Acidophilus* LA14 (*L. Acidophilus*), and *Bifidobacterium Bifidum* BGN4 (*B. Bifidum*), constituents of the commercial blend Acronelle^®^ (Bromatech S.r.l., Milan, Italy). We aimed to compare TDO 2 levels in extracellular vesicles (EVs) from plasma before and after the treatment regimen. In general, EVs are small membrane-bound structures released by cells into their external environment. These vesicles play important roles in intercellular communication, transferring various biomolecules, such as proteins, lipids, and nucleic acids, between cells. Thus, EVs are involved in cell-to-cell information transfer and also participate in cellular responses against stress [22,23]. Since EVs can mediate the transfer of specific molecules, they could play a role in intercellular transmission in the pathogenesis of human diseases [22,23]. Based on these preliminary in vivo findings, we aimed to further investigate the potential benefits of a probiotic mix containing the same strains found in Acronelle^®^. To achieve this, we used an in vitro model of HT29 colorectal carcinoma cells, which serve as a well-established and widely utilized model for investigating various aspects of the human intestinal tract, particularly the intricate interactions between the host and microbial components [24,25,26]. We treated HT29 cells with the probiotics mix, either alone or in combination with hydrogen peroxide, for 24 h (probiotics mix 24 h, H_2_O_2_ 24 h, and probiotics mix and H_2_O_2_ 24 h synchronously). Hydrogen peroxide is frequently utilized as a stress-inducing agent in numerous in vitro models and is recognized for its ability to disrupt the epithelial barrier [27,28]. Additionally, we explored different treatment timings to assess the preventive effects of probiotics against hydrogen peroxide-induced damage: probiotics mix for 2 h followed by H_2_O_2_ for 22 h, and vice versa. The results of this study suggest that the probiotics mix not only provides protection against stress and restores the epithelial barrier but also activates the tryptophan pathway via EVs. This activation may extend the regulation of host homeostasis, potentially offering protection against neurodegenerative and neuropsychiatric disorders, including depression, at the level of the central nervous system. To the best of our knowledge, this study represents the first demonstration that the hypothesized gut–brain axis can be mediated by nanovesicles, facilitating the transfer of mediators to the central nervous system and thereby influencing its function.

## 2. Materials and Methods

### 2.1. Antibodies

Full details of the primary antibodies used are reported in Table 1.

### 2.2. Participant Recruitment

A total of 20 subjects with dysbiosis were considered for this study and received 2 capsules of Acronelle^®^ (Bromatech S.r.l., Milan, Italy), containing 3 probiotic strains (*L. salivarius* strain LS33, *L. acidophilus* strain LA14, and *B. bifidum* strain BGN4) orally twice a day after meals for 60 days. Plasma has been sampled from subjects before and after the assumption period. All subjects gave written informed consent that was validated and approved by the ethical committee of the Policlinico “Paolo Giaccone” University of Palermo, Palermo, Italy (ethics committee approval number 03/2023).

### 2.3. Plasmatic EV Purification and Characterization 

Plasma from blood was processed for separation by centrifugation at 1800× *g* for 30 min at 22 °C, and plasma samples were stored at −80 °C until processing. EVs were isolated from plasma by several steps of differential ultracentrifugation and ultrafiltration as described before [29]. The EV pellet was collected, washed once in PBS, and then suspended in 100 μL of PBS for morphological analyses (DLS, dynamic light scattering, and STEM, scanning transmission electron microscopy) or a 70 µL RIPA buffer (50 mM Tris/HCl, 150 mM NaCl, 1% NP-40, 1 mM EGTA, supplemented with Protease Inhibitor Cocktail (Sigma-Aldrich, St. Louis, MO, USA)) to detect the presence of typical exosome markers.

#### 2.3.1. DLS

The size distribution of the vesicles was determined by DLS experiments. Samples were pipetted after thawing and were centrifuged at 1000× *g* for 10 min at 4 °C to remove eventual aggregates. The supernatant was placed at 20 °C in a thermostated cell compartment of a Brookhaven Instruments BI200-SM goniometer (Holtsville, NY, USA), equipped with a He-Ne laser (JDS Uniphase 1136P, AERI LTD, Bath, UK) tuned at 633 nm and a single-pixel photon counting module (Hamamatsu C11202-050, Hamatsu, Massy Cedex, France). Measurements have been performed as previously described [30]. The size distribution Pq(σ) is calculated by assuming that the diffusion coefficient distribution is shaped as a Schultz distribution, which is a two-parameter asymmetric distribution, determined by the average diffusion coefficient ^−^D (σz is the diameter corresponding to ^−^D) and the polydispersity index PD [30]. The analysis was performed using two species: the former to consider the contribution from small particles around 20 nm or less (proteins or small molecules) and the latter for the contribution assigned to vesicles. Only in particular cases was a third contribution added to consider the presence of residual diffusing objects around 1 µm.

#### 2.3.2. STEM 

EVs obtained by ultracentrifugation were resuspended in PBS and then deposited onto formvar nickel grids. Subsequently, the grid-mounted preparations were stained with 1% uranyl acetate for 5 min, followed by treatment with Reynolds’ solution for another 5 min. Finally, they were rinsed eight times in distilled water for 2 min each. Following this procedure, the grids were prepared for electron microscopy (FEI-Thermo Fisher Versa 3D scanning electron microscope equipped with a retractable STEM detector; Therni Fisher, Waltham, MA, USA).

#### 2.3.3. Western Blot Analysis

Western blotting analyses of vesicles were performed as previously described [29]. Briefly, 60 µg of vesicle lysates were denatured under reducing conditions by boiling for 3 min at 95 °C in 50 mM Tris HCl (pH. 6.8), 1% SDS, 2% β-mercaptoethanol, and 0.01% bromophenol blue. Proteins were separated by 12% SDS-PAGE along with a molecular weight marker (Bio-RAD laboratories, Segrate Milan, Italy) and then transferred by electrophoresis to nitrocellulose membranes (Millipore, Bedford, MA, USA). After transfer, all membranes were stained with Ponceau S to verify the quality of transfer and loading similarity. After blocking the solution with 5% albumin bovine serum (Sigma Aldrich, Darmstadt, Germany) in Tris-Buffered Saline (20 mM Tris, 137 MM NaCl, pH 7.6) with 0.05% Tween-20 (T-TBS), the membranes were incubated in 0.05% BSA in T-TBS with a CD81 primary antibody overnight at room temperature (Table 1). Blots were washed in T-TBS and incubated for 1 h in 0.05% BSA in T-TBS with a secondary antibody Ig (anti-mouse IgG, 1:10,000, GTX26808, Gene Tex, Irvine, CA, USA; anti-rabbit IgG, 1:20,000, 12348, Millipore, Burlington, MA, USA). Protein bands were visualized using the enhanced chemiluminescence detection system, Western Blotting Detection Reagent (Amersham Biosciences, GE Healthcare Life Science, Milan, Italy), according to the manufacturer’s instructions. The protein levels were normalized for GAPDH, and the data were evaluated and quantified using the NIH Image J 1.40 analysis program (National Institutes of Health, Bethesda, MD, USA). Each experiment was performed in triplicate. 

### 2.4. In Vitro Study 

#### 2.4.1. Bacterial Strains and Culture Conditions

The probiotic strains tested in this study were *L. salivarius* strain LS33, *L. acidophilus* strain LA14, and *B. bifidum* strain BGN4, all of which are present in the probiotic blend Acronelle^®^ taken from subjects enrolled in this study. We specifically decided to test these strains in vitro by implementing a model of intestinal epithelium subjected to stress because these strains are the same as those ingested by subjects with irritable bowel syndrome (IBS) and subtype diarrhea (IBS-D). The probiotic strains, hereafter called the “probiotics mix”, were provided by Bromatech S.r.l., Milan, Italy, and were separately cultured in de Man–Rogosa–Sharpe (MRS) broth medium (37 °C) for 18–24 h on a shaker at 250 rpm. The next day, optical density (OD) measurement at 600 nm was performed to collect the adequate CFU of each strain.

#### 2.4.2. Cell Culture 

The human colon adenocarcinoma cell line (HT29) was grown in Roswell Park Memorial Institute (RPMI) 1640 medium (Gibco-Thermo Fisher Scientific, Waltham, MA, USA) supplemented with 10% fetal bovine serum (FBS), 100 U/mL penicillin, 100 g/mL streptomycin, and 2 mM L-glutamine in a humidified 37 °C atmosphere containing 5% CO_2_. The cultures were fed with fresh medium every alternate day and subcultured following enzymatic digestion using 0.25% Trypsin–EDTA solution (Sigma, USA) at 37 °C.

#### 2.4.3. Treatments of Cells

HT-29 cell suspension was seeded in a 96-well microplate at a density of 7 × 10^3^ cells for cell viability assay and in T25 flasks at a density of 1 × 10^6^ cells for Western blotting analyses. The viability rate was measured by MTT (3-(4,5-dimethylthiazol-2-yl-2,5-diphenyl tetrazolium bromide) assay (Sigma-Aldrich) as previously reported [31]. Briefly, after seeding, cells were starved for 24 h and then treated separately with 10^9^ CFU/mL serial dilution of each strain to assess the max inhibition rate. Cells were also challenged with H_2_O_2_ for 24 h. After the above treatments, cells were incubated with 20 μL of fresh MTT solution (11 mg/mL), and incubation was prolonged for 2 h at 37 °C and 5% CO_2_. The reaction was stopped replacing the medium with 100 μL of DMSO. After solubilization, the absorbance of the product was read at 570 and 690 nm by an ELISA reader. Each condition was tested six times, and results were analyzed for statistical significance. Based on MTT results, the conditions chosen for all subsequent experiments were mixed to create a probiotics mix composed of 1 × 10^8^ CFU/mL for the LS33 strain, 4 × 10^8^ CFU/mL for the LA14 strain, and 2 × 10^8^ CFU/mL for the BGN4 strain; with 6 different conditions: 1: untreated cells; 2: cells treated with a probiotics mix for 24 h; 3: cells pre-treated with a probiotics mix for 2 h and then with H_2_O_2_ (1 mM) for 22 h; 4: cells treated with H_2_O_2_ for 24 h; 5: cells pre-treated with H_2_O_2_ for 2 h and then with a probiotics mix for 22 h; and 6: cells treated with simultaneously with a probiotics mix and H_2_O_2_ for 24 h [32].

#### 2.4.4. Protein Extraction, Quantification, and Western Blot Analysis 

At the end of the treatments, the cell culture medium of each treatment was collected and stored at −20 °C until subsequent analysis. After treatment, HT29 cells were pelleted and resuspended in a RIPA buffer for Western blot analyses, performed as described above. 

#### 2.4.5. Immunofluorescence Staining and Confocal Microscopy

For immunofluorescence, the cells were placed in eight-well chamber slides, cultured for 24 h according to the treatment, fixed in ice-cold methanol for 30 min, and incubated with an unmasking solution (10 mM trisodium citrate, 0.05% Tween 20, pH 6) for 10 min at room temperature. After rinsing twice with PBS, the cells were blocked with 3% bovine serum albumin (BSA, Sigma Aldrich) in PBS for 30 min at room temperature and incubated in a humidified chamber overnight at 4 with primary antibodies (Table 1). The day after, the cells were washed twice in PBS and were incubated with a fluorescent secondary antibody (anti-rabbit IgG-FITC antibody produced in goat, 1:50, F0382, Sigma-Aldrich, St. Louis, MO, USA; anti-mouse IgG-Atto 488 antibody produced in goat, 1:50, 62197, Sigma-Aldrich). The nuclei were counterstained with DAPI 33342 (1:1000, Sigma-Aldrich, St. Louis, MO, USA) for 15 min at 22 °C. Finally, slides were mounted with cover slips, and images were taken immediately with a Leica Confocal Microscope TCS SP8 (Leica Microsystems, Heidelberg, Germany). Confocal microscopy analysis was performed using the Leica application suite advanced fluorescence software. Staining intensity was quantified by calculating the mean pixel intensity (PI) normalized to the cross-sectional area (CSA) of all cells in five fields per slide. Each experiment was conducted in quadruplicate.

#### 2.4.6. Isolation of Vesicles from Culture Medium

A total of 10 mL of conditioned medium from 2 × 10^6^ HT29 cells were collected after 24 h of treatment in serum-free medium and centrifuged (800× *g* for 10 min) at 4 °C to eliminate cells and debris. Vesicles have been isolated using the total exosome isolation kit (Invitrogen, Life Technologies, Carlsbad, CA, USA), following the manufacturing instructions. Briefly, cell media was centrifuged at 2000× *g* for 30 min to remove cells and debris. The reagent was added and mixed to cell-free culture media and after overnight incubation at 2 °C to 8 °C, and the suspension was centrifuged the samples at 10,000× *g* for 1 h at 2 °C to 8 °C. The pellet containing vesicles was then suspended in 100 μL of PBS or a 70 µL RIPA buffer. Outer membrane vesicles (OMVs) were isolated from the culture MRS broth medium of each bacterial strain. The medium was centrifuged at 13,000× *g* for 20 min at 4 °C to remove cell debris. Then, the supernatant was ultracentrifuged at 110,000× *g* for 2 h, and then the OMV pellet was collected and resuspended in 100 μL of PBS for biophysical characterization [33]. The morphological features of vesicles from HT29 cells and OMVs were assessed as described above.

### 2.5. Statistical Analyses

Assays were carried out in triplicate in at least three independent experiments, data were expressed as the mean ± S.D, and the level of statistical significance was set at *p* < 0.05. All statistical analysis was performed with GraphPad PrismTM 8.0 (GraphPad Software Inc., San Diego, CA, USA) through analysis of variance (ANOVA) followed by Bonferroni post hoc tests for multiple comparisons. Student’s *t*-test was used to identify statistically significant differences between the two groups.

## 3. Results

### 3.1. TDO 2 Is Secreted in Blood Circulation through EVs

The existence of a well-established gut–brain axis, mediated by the neuroendocrine system, nervous system, and circulatory system, has long been acknowledged [34,35]. Numerous metabolites, including tryptophan, actively contribute to regulating the overall organism and brain homeostasis within this axis. Recently, significant attention has been directed towards the role of tryptophan metabolism as a metabolite through which gut microbes affect the metabolic pathways of the host [36,37]. Tryptophan is preferentially converted to kynurenine by enzymes as Indoleamine-pyrrole 2,3-dioxygenase (IDO) and Tryptophan 2,3-dioxygenase (TDO 2), which may be regulated directly or indirectly by the gut microbiome. While tryptophan, as an essential amino acid and precursor for the neurotransmitter serotonin (5-HT), has garnered significant attention, the mechanisms by which gut bacteria regulate circulating tryptophan concentrations remain unclear [38]. Here, we assessed the levels of TDO 2 in plasmatic extracellular vesicles of subjects with IBS, before and after the administration of 60-day Acronelle^®^ (Bromatech S.r.l., Milan, Italy), as part of our endeavor to demonstrate the existence of an alternative gut–brain axis, hitherto only hypothesized but never substantiated, involving extracellular vesicles. From April 2023 to December 2023, we recruited 20 subjects (aged between 30 and 66 years old, 12 males and 8 females) with a diagnosis of IBS-D according to the Rome IV criteria, which lasts longer than 15 days [39,40,41]. Subjects with organic diseases, such as chronic diseases, including cancers, inflammation or surgical history in the alimentary tract, or serious systematic dysfunctions, were excluded. During the treatment period, patients followed a diet based on traditional dietary advice (TDA) developed by the National Institute for Health and Care Excellence (NICE) and the British Dietetic Association (BDA), which is recognized as a primary dietary approach by the recent Italian Guidelines for the Management of Irritable Bowel Syndrome in 2023 [42,43]. As reported by the subjects in our study group, Acronelle^®^ (Bromatech S.r.l., Milan, Italy) conferred significant improvements in the Gastrointestinal Symptom Rating Scale-IBS (GSRS-IBS) and Bristol Stool Form Scale (BSFS), further confirming the effectiveness of probiotic supplementation in alleviating symptoms of IBS [44,45].

**Figure 1:**
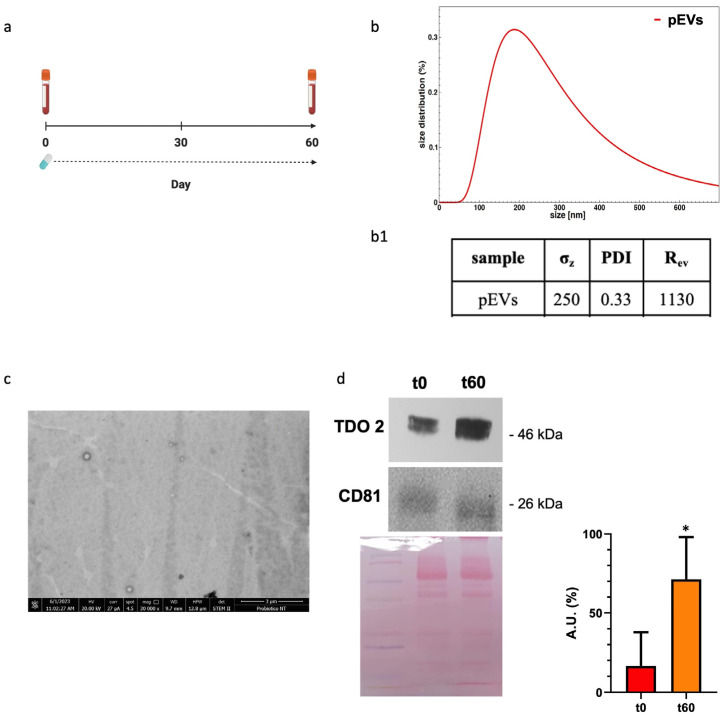
Characterization and TDO 2 content of EVs isolated from the plasma of subjects with dysbiosis. (**a**) Timeline of the blood draws conducted on subjects who took Acronelle^®^ for 60 days. (**b**) Size and size distribution of vesicles determined by dynamic light scattering (DLS) experiments. The red curve represents the size distribution of plasmatic EVs (pEVs). (**b1**) Parameters of biophysical characterization of vesicles by DLS (σz: z-averaged hydrodynamic diameter; PDI: polydispersity index; Rev: Rayleigh ratio of vesicle population). (**c**) Electron micrography of pEVs. (**d**) Representative Western blot and relative histograms showing the presence and levels of TDO 2 in pEVs, which show higher levels of TDO 2 in the plasma of patients with dysbiosis after 60 days (t60) of probiotics mix assumption compared with those obtained from the same subjects (t0). The image shows CD81 as a typical EV marker and Ponceau staining as a loading control. * *p* < 0.05.

We analyzed our EV preparations from plasma by DLS and electron microscopy and found that the size and the morphology of the vesicles obtained were typical of EVs (Figure 1(b(b1),c)). Western blotting showed that the plasma EV preparations expressed CD81, a typical exosome marker (Figure 1d, bottom row). TDO 2 was detected in the exosomes from the plasma of subjects with dysbiosis before the treatment, and its levels were higher after 60 days of probiotics mix administration (Figure 1d, top row). 

### 3.2. The Probiotics Mix of LS33, LA14, and BGNA4 Strains Has Cytoprotective Properties on HT29 Cells

**Figure 2:**
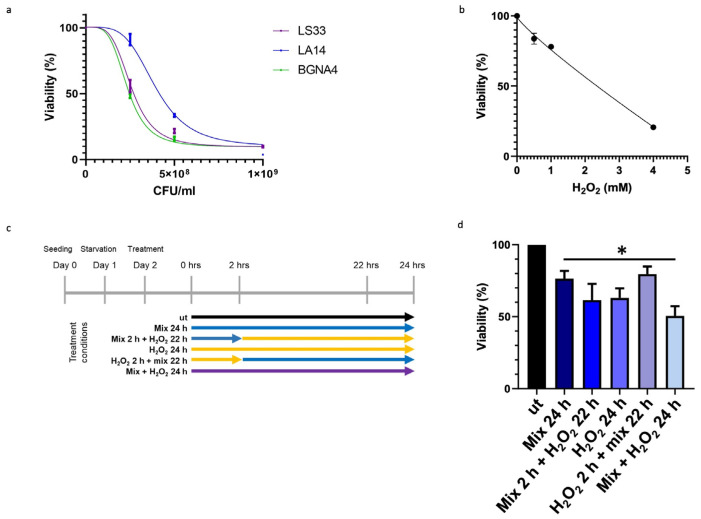
Effect of probiotics on the viability of HT29 measured by the MTT assay. (**a**) Effect on the viability of HT29 treated with LS33, LA14, and BGNA4. Nonlinear regression and IC_50_ calculations were performed using GraphPad Prism 8 software (GraphPad Software). IC_50_ LS33: 2.7 × 10^8^ CFU/mL; IC_50_ LA14: 5.3 × 10^8^ CFU/mL; IC_50_ BGNA4: 3.3 × 10^8^ CFU/mL. Values are presented as means ± S.D. (n = 3). (**b**) Nonlinear regression and IC_50_ calculations of the effect on the viability of HT29 treated with H_2_O_2_ and IC_50_ calculation. IC_50_ H_2_O_2_: 2135 mM. Values are presented as means ± S.D. (n = 4). (**c**) Timeline of treatments: ut: untreated cells; mix 24 h: cells treated with a probiotics mix for 24 h; mix 2 h + H_2_O_2_ 22 h: cells pretreated with a probiotics mix for 2 h and then with H_2_O_2_ for 22 h; H_2_O_2_ 24 h: cells treated with H_2_O_2_ for 24 h; H_2_O_2_ 2 h + mix 22 h: cells pretreated with H_2_O_2_ for 2 h and then with a probiotics mix for 22 h; mix + H_2_O_2_ 24 h: cells treated simultaneously with a probiotics mix and H_2_O_2_ for 24 h. (**d**) Cytoprotective effect of the probiotics mix on H_2_O_2_-exposed HT29. The data represent the mean ± S.D. (n = 8). * <0.05 vs. UT.

To ascertain that the observed elevation in TDO 2 levels within vesicles isolated from the plasma of subjects with IBS resulted from probiotic administration, we employed an in vitro approach using HT29 cells. Specifically, we developed a cellular stress model mediated by H_2_O_2_, in which cells were subsequently treated with the same probiotic strains present in Acronelle^®^ (Bromatech S.r.l., Milan, Italy), as ingested by individuals with IBS-D. To set the treatment conditions of the probiotics mix and H_2_O_2_ to be used on HT29 cells, we measured cell viability by an MTT assay. Cells were treated with increasing concentrations of LS33, LA14, and BGNA4 strains separately and with H_2_O_2_ for 24 h within the range of 0–1 × 10^9^ CFU/mL and 0–5 µM, respectively (Figure 2a,b). Then, HT29 cells were treated following the timeline of treatments summarized in Figure 2c. The probiotics mix had a cytoprotective effect on H_2_O_2_-exposed HT29 cells. When the HT29 cells were pretreated with H_2_O_2_ for 2 h before being exposed to the probiotics mix for 22 h, cell viability increased significantly (*p*-value < 0.05) compared with that of the groups (Figure 2d).

### 3.3. Anti-Stress Effect of Probiotics against Oxidative Stress Induced by H_2_O_2_ in HT29 Cells

**Figure 3:**
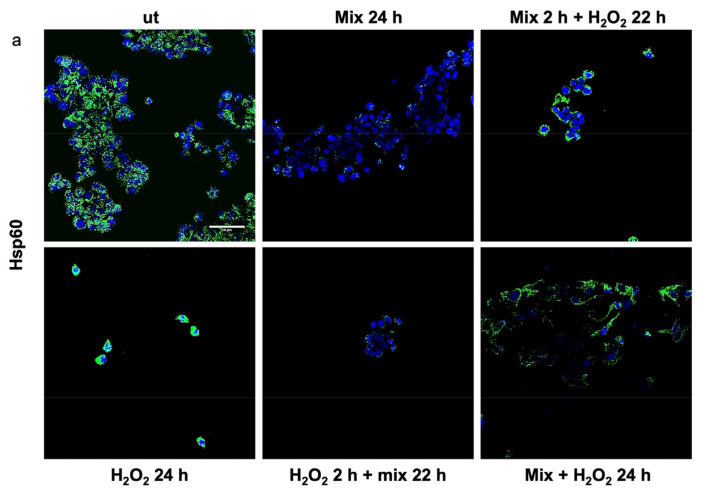

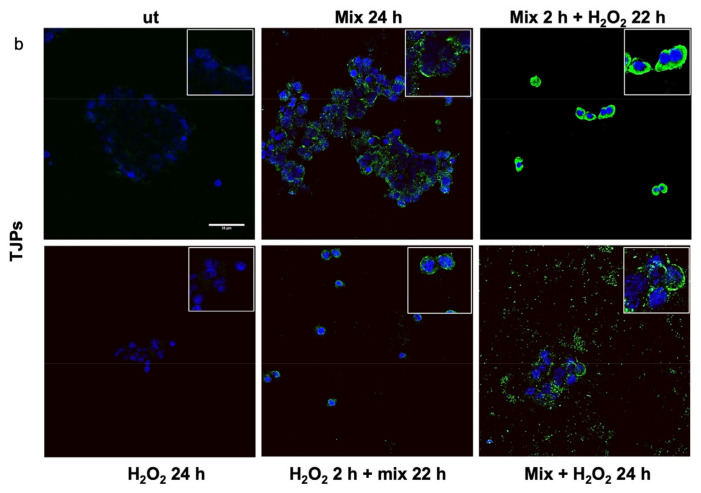
Anti-stress effect of probiotics against oxidative stress induced by H_2_O_2_ in HT29. Representative images captured for Hsp60 (green) (**a**) and tight junction protein (TJP) (green) (**b**) detection using immunofluorescence. Nuclei were counterstained with DAPI (blue). The observation using confocal microscopy confirmed that the probiotic treatment reduced stress induced by H_2_O_2_ in HT29 and restored the epithelial barrier. Scale bars = 50 μm. ut: untreated cells; mix 24 h: cells treated with a probiotics mix for 24 h; mix 2 h + H*_2_*O*_2_* 22 h: cells pretreated with a probiotics mix for 2 h and then with H_2_O_2_ for 22 h; H_2_O_2_ 24 h: cells treated with H_2_O_2_ for 24 h; H_2_O_2_ 2 h + mix 22 h: cells pretreated with H_2_O_2_ for 2 h and then with a probiotics mix for 22 h; mix + H_2_O_2_ 24 h: cells treated simultaneously with a probiotics mix and H_2_O_2_ for 24 h.

Previous studies have shown that H_2_O_2_ increases Hsp60 protein levels [46] and disrupts TJPs [47]. Here, we evaluated the anti-stress activity after treating H_2_O_2_-exposed HT29 cells with the probiotics mix by assessing the level of Hsp60 and TJP proteins. The 24 h H_2_O_2_-exposed cells showed higher levels of Hsp60 (Figure 3a). Conversely, the Hsp60 levels in HT29 treated with the probiotics mix for 24 h were significantly lower than in control cells (Figure 3a). Moreover, compared with the control, the Hsp60 levels were reduced in cells after exposure to the probiotics mix for 2 h and then H_2_O_2_ for 22 h, in cells pre-treated with H_2_O_2_ for 2 h and then a probiotics mix for 22 h, and in cells treated simultaneously with a probiotics mix and H_2_O_2_ for 24 h (Figure 3a). To assess the protective impact of probiotics against the H*_2_*O*_2_*-induced disruption of TJPs, we examined the distribution within cells and the levels of TJPs’ proteins through immunofluorescence staining and analysis using confocal microscopy. Results show that H_2_O_2_ treatment resulted in a reduction of TJPs (Figure 3b). This reduction was attenuated by treating cells with probiotics mix (Figure 3b).

### 3.4. Probiotics Treatment Increases TDO 2 and Hsp90 Protein Levels in HT29 Cells

**Figure 4:**
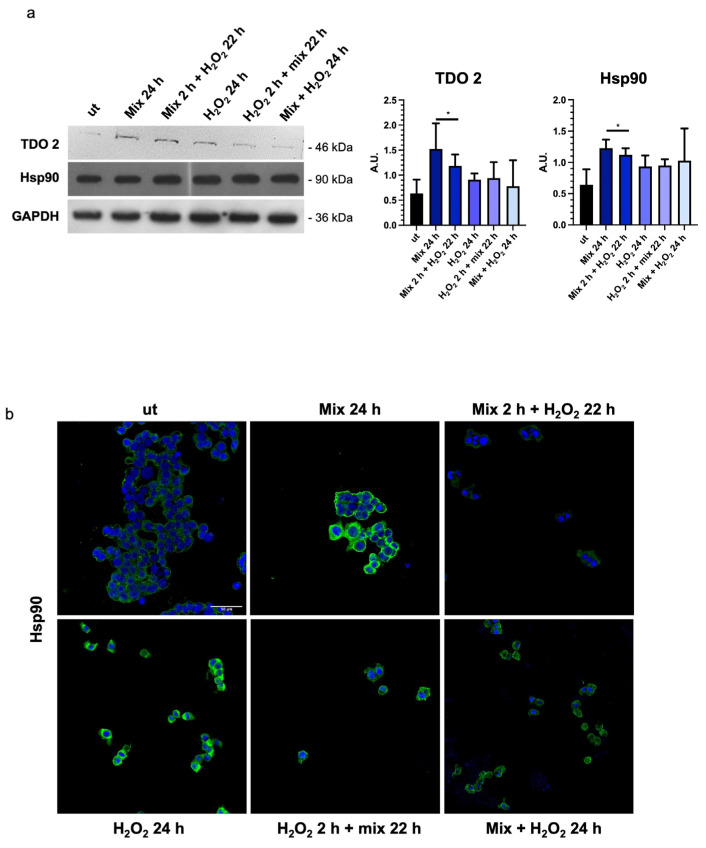
Probiotics treatment increases TDO 2 and Hsp90 protein levels. (**a**) Representative blots and histograms show that the probiotics mix treatment induced an increase in TDO 2 and Hsp90 protein levels in HT29 cells. GAPDH was used as an internal control. The histograms show the TDO 2/GAPDH and Hsp90/GAPDH levels (mean ± SD) expressed as arbitrary units (AUs). The results are representative of three independent experiments (* *p* < 0.0001, compared to ut). (**b**) Representative images of immunofluorescence detecting Hsp90 (green), confirming the data obtained by the Western blot analysis. Nuclei were counterstained with DAPI (blue). Scale bar = 50 μm. ut: untreated cells; mix 24 h: cells treated with a probiotics mix for 24 h; mix 2 h + H_2_O_2_ 22 h: cells pretreated with a probiotics mix for 2 h and then with H_2_O_2_ for 22 h; H_2_O_2_ 24 h: cells treated with H_2_O_2_ for 24 h; H_2_O_2_ 2 h + mix 22 h: cells pretreated with H_2_O_2_ for 2 h and then with a probiotics mix for 22 h; mix + H_2_O_2_ 24 h: cells treated simultaneously with a probiotics mix and H_2_O_2_ for 24 h.

In intestinal cells, during the stress response, tryptophan is converted to kynurenine by stress-sensitive enzymes, such as TDO 2. Therefore, we decided to evaluate the levels of TDO 2 in H_2_O_2_-exposed HT29 cells after treatment with the probiotics mix. We observed that TDO 2 protein levels were significantly increased in cells treated with the probiotics mix for 24 h (Figure 4a). 

In addition, to explore the activation of the tryptophan-mediated signaling pathway leading to the modulation of barrier function and stress response through the transcription factor aryl hydrocarbon receptor (AHR) [48], we investigated changes in the molecular chaperone Hsp90, which is involved in nuclear translocation of AHR [49]. A significant increase in Hsp90 protein levels was observed in HT29 cells treated with the probiotics mix for 24 h, the probiotics mix for 2 h, and H_2_O_2_ for 22 h (Figure 4a,b).

### 3.5. The Probiotics Mix Modulates the Expression of Serotonin Receptors in HT29 Cells

**Figure 5:**
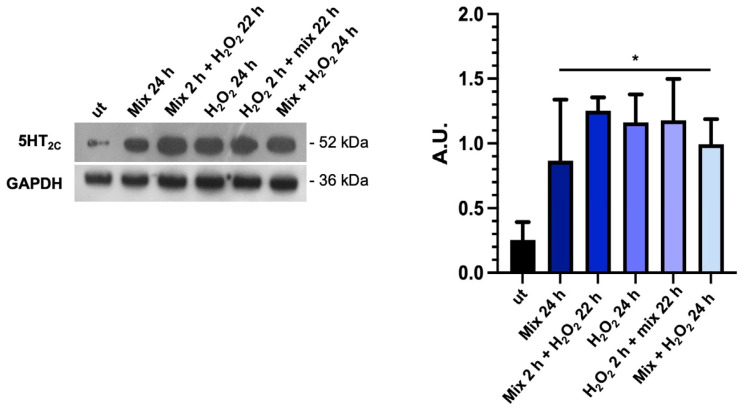
The probiotics mix modulates the expression of serotonin receptors. Western blot images of 5-HT_2C_ protein levels and relative histograms of HT-29 cells treated with the probiotics mix and H_2_O_2_. GAPDH was used as an internal control, and results are representative of three independent experiments * *p* < 0.05. ut: untreated cells; mix 24 h: cells treated with the probiotics mix for 24 h; mix 2 h + H_2_O_2_ 22 h: cells pretreated with a probiotics mix for 2 h and then with H_2_O_2_ for 22 h; H_2_O_2_ 24 h: cells treated with H_2_O_2_ for 24 h; H_2_O_2_ 2 h + mix 22 h: cells pretreated with H_2_O_2_ for 2 h and then with a probiotics mix for 22 h; mix + H_2_O_2_ 24 h: cells treated simultaneously with a probiotics mix and H_2_O_2_ for 24 h.

Considering that the tryptophan metabolism and TDO 2 activity are likely important factors in the production and availability of serotonin (5-HT), which is important in several gut functions, such as motility and secretion [50], we investigated the expression of 5-HT_2C_ by Western blot analysis. We found that basal 5-HT_2C_ receptor levels were low in untreated HT29 cells; conversely, they increased significantly under all treatment conditions with the probiotics mix and H_2_O_2_ (Figure 5). The increase in 5-HT_2C_ protein levels in the presence of H_2_O_2_ alone could be a defense mechanism of cells aimed against stress since there is a role for 5-HT in anti-stress response. The significance of these data will need to be further explored [51]. 

### 3.6. Probiotics Treatment Increases TDO 2 and Hsp90 Protein Levels in EVs Isolated from HT29

**Figure 6:**
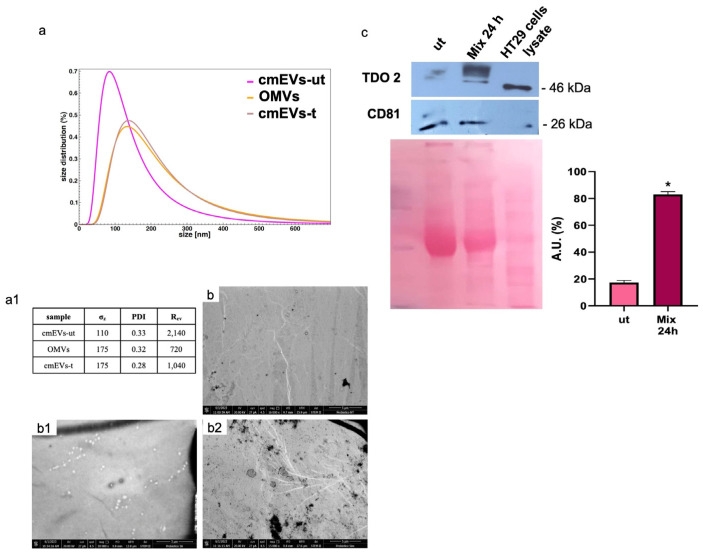
Characterization and TDO 2 content of EVs isolated from conditioned medium of HT29. (**a**) Size and size distribution of vesicles determined by dynamic light scattering (DLS) experiments. The red curve represents the size distribution of plasmatic EVs (pEVs); the pink curve shows conditioned medium EVs (cmEVs-ut) of untreated HT29; the ocher curve shows outer membrane vesicles (OMVs); the gray curve shows conditioned medium EVs of HT29 treated with the probiotics mix of cmEV-treated cells (cmEVs-t). (**a1**) Parameters of the biophysical characterization of vesicles by DLS (σz: z-averaged hydrodynamic diameter; PDI: polydispersity index; Rev: Rayleigh ratio of vesicle population). (**b**,**b1**,**b2**) Electron micrography of cmEVs-ut, cmEVs-t, and OMVs, respectively. (**c**) Representative Western blot and relative histograms showing the presence and levels of TDO 2 in HT29-derived EVs. The higher levels of TDO 2 in EVs isolated from cell medium after treatment with the probiotics mix compared with those obtained from untreated cells are visible. The image shows CD81 as a typical EV marker and Ponceau staining as a loading control. * *p* < 0.05. ut: untreated cells; mix 24 h: cells treated with the probiotics mix for 24 h.

We isolated vesicles from both cell culture medium and bacterial broth. Morphological characterization confirmed that EVs from untreated cell medium (cmEVs-ut) exhibited typical nanovesicle sizes (20–150 nm) (Figure 6(a(a1),b(b1))), while OMVs were larger, ranging between 100 and 300 nm (Figure 6(a(a1,b2))). We evaluated TDO 2 levels in EVs isolated from both untreated and probiotics-treated HT29 cells for 24 h, revealing a significant increase in TDO 2 levels following treatment (Figure 6c).

## 4. Discussion

Emerging evidence suggests that gut microbiota has important value for maintaining the body’s homeostasis. It participates in digestion as part of the intestinal barrier, where it contributes to the prevention of pathogenic colonization, promotes mucus production and regeneration of intestinal epithelium cells, and participates in the synthesis of neuroactive substances [11]. Furthermore, the bowel mucosa establishes multiple connections with other anatomical districts through nanovesicular trafficking [9]. Consequently, alterations in the composition of the gut microbiota led to a condition known as dysbiosis, resulting in damage to the epithelial barrier and impaired nutrient absorption. Dysbiosis has also been associated with gastrointestinal disorders, including IBS. Due to brain–gut interactions, this condition can adversely affect psychological well-being, leading to mental health conditions [52]. It has been well established that microbiome dysbiosis is an indicator or potentially exacerbates anxiety- and depression-like behaviors. This is supported by studies indicating that mice subjected to direct stress exhibit reduced levels of gut *Lactobacillus*. Among metabolites involved, it seems that the IFNγ, an inducer of tryptophan conversion to kynurenine produced in response to *Lactobacillus*, can be sufficient to provide resilience in response to environmental stressors [53,54]. Furthermore, the largest contributor among metabolites that the gut microbiota uses to signal the human brain is tryptophan and its precursors [55]. Tryptophan can be absorbed from the gut and become available in circulation for distribution to target distant sites, such as the central nervous system. Otherwise, it can be metabolized by the gut microbiota and gastrointestinal cells, generating a range of metabolites with diverse biological activities [56]. This prompted us to study protein levels of TDO 2, one of the enzymes responsible for the conversion of tryptophan to kynurenine. Interestingly, among subjects with dysbiosis, TDO 2 is present in plasma nanovescicles, and its levels are higher after 60 days of Acronelle^®^ (Bromatech S.r.l., Milan, Italy) assumption (Figure 1). Very few studies to date have demonstrated the presence of TDO 2/IDO in EVs obtained from patients with dysbiosis or inflammatory diseases [57,58,59,60] and, to the best of our knowledge, changes of TDO 2 levels in plasmatic EVs before and after probiotics treatment have not been described previously. Our results indicate that by plasmatic EVs, the microbiota can drive signals to the brain by inducing the modulation of neurotransmitters and their receptors. We validated our results in vivo using an in vitro stress model. Specifically, we treated HT29 cells with the probiotics mix, simulating the conditions of individuals with IBS who consumed Acronelle^®^. The probiotics mix exhibited cytoprotective properties against H_2_O_2_-induced stress, with significant increases in cell viability observed when cells were pretreated with H_2_O_2_ for 2 h followed by the probiotics mix for 22 h (Figure 2d). Variations in the composition of the gut microbiota may be due to various environmental factors, such as aging, inflammation, and chronic stress [61]. In cellular responses to environmental stressors, a particular class of proteins, Hsps, are constitutively activated to lead to the restoration of cellular homeostasis. Among them, Hsp60 is a mitochondrial chaperonin significantly increased under stress conditions and is involved in the recovery and protection from cell damage [46,62]. In this work, we confirmed the cytoprotective effect of probiotics in the intestinal mucosa by evaluating the levels of Hsp60 protein and TJPs in HT29 cells. Previous studies have demonstrated that intestinal mucosa stress is associated with epithelial barrier injury [63] and suggest that a key role in the mechanism of tissue damage could be played by Hsp60, which works as an immune system signal factor [64]. Our group demonstrated that in patients with ulcerative colitis, treatment with either 5-aminosalicylic acid alone or in combination with a probiotic was effective in reducing symptoms, and this amelioration was associated with the reduction of both inflammation and Hsp60 levels [64]. More recently, we highlighted the benefits of L. fermentum in a mice model of oxidative stress damage and inflammation induced by chronic alcohol intake. The probiotic’s protective and anti-inflammatory effects in the intestine, mediated by Hsp60 expression, seem to extend to the cerebellum, likely due to the maintenance of intestinal barrier integrity [65]. In this work, our in vitro data revealed that treatment with a probiotics mix can protect cells from H_2_O_2_-induced stress, reducing levels of the stress-inducible protein Hsp60, as shown by immunomorphological analysis of Hsp60 levels (Figure 3a). Furthermore, treatment with a probiotics mix can preserve intestinal integrity and barrier function by the restoration of expression and redistribution of TJPs (Figure 3b). This effect is relevant in HT29 cells, where the TJPs are not very compact [24]. In fact, HT29 is grown as a multilayer of non-polarized cells under normal growth conditions with a relatively low expression of tight junctions, as shown in immunofluorescence images (Figure 3b). In our experiments, HT29 treated with H_2_O_2_ for 24 h showed a further decrease in TJP levels, whereas subsequent (H_2_O_2_ 2 h + mix 22 h) and simultaneous treatment (H_2_O_2_ + mix 24 h) with a probiotics mix restored their levels and localization. Notably, pre-treatment with the probiotics mix for 2 h seemed to have a protective effect against subsequent H_2_O_2_-induced stress (mix 2 h + H_2_O_2_ 22 h). The mechanism by which several probiotic strains improve barrier function could be due to a redistribution of TJPs between the cytoplasm and membrane and a post-translational modification, such as phosphorylation [66]. We studied TDO 2, the enzymes responsible for the conversion of tryptophan to kynurenine, and Hsp90, a molecular chaperone involved in the translocation of the AHR (aryl hydrocarbon receptor) factor into the nucleus, where it activates anti-inflammatory response [67]. In the current study, we found that TDO 2 and Hsp90 protein levels are increased in cells treated with the probiotics mix (Figure 4). 

Particularly, TDO 2 is significantly increased after treatment with the probiotics mix for 24 h and mix 2 h + H_2_O_2_ 22 h compared with the control (Figure 4a), while Hsp90, which we evaluated by Western blot and immunofluorescence analyses, increased in all conditions tested (Figure 4a,b). The increased protein level of TDO 2 and Hsp90 in cells treated with probiotics could be correlated with the increase in tryptophan availability. Tryptophan metabolism in the gut also occurs through the serotonin (5-hydroxytryptamine) pathway; therefore, we decided to explore the expression of the 5-HT_2C_ receptor in HT-29 cells treated with different combinations of the probiotics mix and H_2_O_2_. We observed an increase in 5-HT_2C_ receptors in all conditions tested, which we supposed may be due to the overexpression of the enzyme TDO 2, which transforms most of the tryptophan into kynurenine, subtracting it from the 5-HT pathway. Consequently, cells might try to retrieve the uptake of serotonin from the extracellular environment by increasing its receptor [68]. To understand whether the increase in TDO2 levels in EVs isolated from plasma of IBS patients treated with Acronelle^®^ (Bromatech S.r.l., Milan, Italy) was related to the treatment and to confirm our in vivo data, we isolated EVs from HT29 cells treated with the probiotics mix for 24 h. Remarkably, TDO2 levels were higher in EVs from treated cells compared to the control (Figure 6). 

Although this study provided very interesting findings on the role of nanovesicles in mediating the gut–brain axis and their potential impact on neurological disorders, it may be subject to limitations. Firstly, our investigation focused solely on assessing the effects of a specific probiotic mix containing *L. Salivarius* LS33, *L. Acidophilus* LA14, and *B. Bifidum* BGN4 on the levels of TDO 2 in nanovesicles isolated from the plasma of subjects with intestinal malabsorption disorders and chronic diarrhea. While our findings offer valuable insights into the potential therapeutic benefits of this probiotic mix, they may not be generalizable to other probiotic formulations. Future studies may benefit from exploring additional mechanisms underlying the interaction between probiotics and host physiology to provide a more comprehensive understanding of probiotic-mediated effects on human health. Moreover, while our in vitro experiments using HT29 colorectal carcinoma cells propose valuable insights into the potential cellular mechanisms underlying probiotic action, they may not fully replicate the complex microenvironment of the human intestine. Therefore, future studies employing more physiologically relevant models, such as organoids or animal models, may be warranted to validate and extend our findings.

## 5. Conclusions

**Figure 7:**
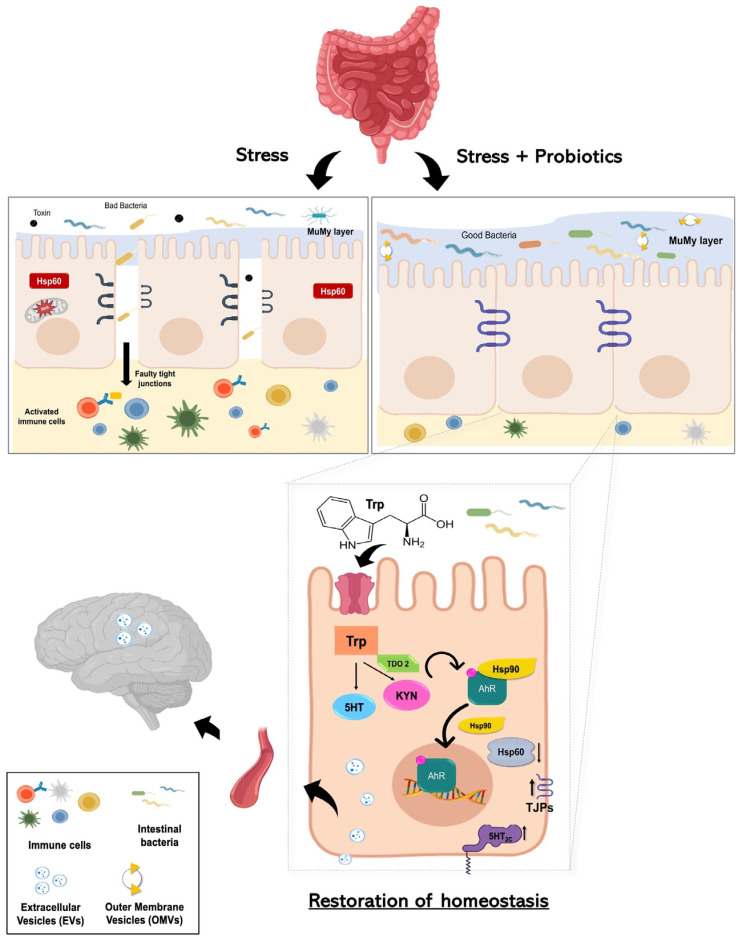
Working hypothesis. Summarizing our working hypothesis, we assume that treatment with the probiotics mix induces an increase in the metabolism of the aminoacidic tryptophan as a precursor for the synthesis of kynurenine. The latter is a product metabolized by the enzyme TDO2. Kynurenine binds AhR, which acts as a ligand-dependent transcription factor capable of controlling complex transcriptional processes. AhR is a cytoplasmic transcription factor that is normally found in an inactive form bound to the co-chaperone Hsp90 and can be activated by various indole derivatives, including kynurenine. Upon binding, the Hsp90 chaperone dissociates and AhR heads into the cell nucleus, acting in favor of the host homeostasis. The probiotics mix acts on cell stress, inducing a reduction of Hsp60 level in H_2_O_2_-exposed cells, and has a positive effect on intestinal permeability, increasing the expression of junctional proteins. Furthermore, following the complex two-way communication system that characterizes the gut–microbiota–brain axis, the increased tryptophan metabolism at the intestinal level induced by probiotic intake could lead not only to increased synthesis of kynurenine but also to increased concentration of serotonin by increasing the levels of 5-HT_2C_ and the systemic levels. This can result in a mechanism that can be able to limit stress-related psychiatric disorders. Finally, since plasma-derived EVs and vesicles isolated from HT-29 cells showed an increase in TDO 2 protein levels following probiotic treatment; our data suggest that EVs may extend the regulation of host homeostasis by inducing protection even at the level of the central nervous system against neurodegenerative and neuropsychiatric disorders, including depression.

Although further investigations are required to validate the direct effects of gut microbiota and probiotics mix effectiveness on tryptophan metabolism in dysbiosis, increased knowledge of the role of the gut flora in health and disease and the effect of probiotics formulation could lead to the development of more effective therapeutic options. Of utmost significance, our study provides the first scientific evidence regarding the actual existence of the hypothesized gut–brain axis mediated by nanovesicles—a signaling method distinct from the neural, lymphatic, or conventional blood pathways (Figure 7). This discovery holds considerable implications for neurological health and may overlay the way for novel therapeutic interventions centered around probiotic consumption.

## Figures and Tables

**Table 1:** Details of sources and concentrations of antibodies used in this study.

Primary Antibody	Overview	Western Blotting (WB)	Immunofluorescence (IF)
Tryptophan 2,3-dioxygenase 2 (TDO 2)	SAB2105644 Rabbit Polyclonal Sigma-Aldrich, Darmstadt, Germany	1:500	-
Heat Shock Protein 60 (Hsp60)	Sc-59567 Mouse Monoclonal Santa Cruz Biotechnology, Dallas, TX, USA	1:1000	1:50
Heat Shock Protein 90 α/β (Hsp90)	Sc-13119 Mouse Monoclonal Santa Cruz Biotechnology, Dallas, TX, USA	1:1000	1:50
CD81	Sc-70803 Mouse Monoclonal Santa Cruz Biotechnology, Dallas, TX, USA	1:1000	-
Tight Junction Protein 1 (TJP1)	C82740 Rabbit Polyclonal Sigma-Aldrich, Darmstadt, Germany	-	1:50
5-HT_2C_	Ab-133570 Rabbit Monoclonal Abcam, Cambridge, UK	1:1000	-
Anti-GAPDH	ABS16 Rabbit Polyclonal Sigma-Aldrich, Darmstadt, Germany	1:1000	-

## Data Availability

The original contributions presented in the study are included in the article, further inquiries can be directed to the corresponding author.
